# Addressing Inappropriate Antibiotic Use: Findings From a Point Prevalence Study in a Tertiary Care Hospital

**DOI:** 10.7759/cureus.90711

**Published:** 2025-08-22

**Authors:** Fusun Z Akcam, Begum Pekbay, Onur Unal, Tugce Ertugrul, Fatih Koçluk, Ali Bahadir Uygun, Ayse Hilal Turker

**Affiliations:** 1 Infectious Diseases and Clinical Microbiology, Suleyman Demirel University, Isparta, TUR; 2 Infectious Diseases and Clinical Microbiology, Süleyman Demirel University, Isparta, TUR

**Keywords:** antibiotic stewardship, inappropriate antibiotic use, infectious diseases consultation, point prevalence survey, surgical prophylaxis, tertiary care hospital

## Abstract

Objective

Inappropriate antibiotic use is a significant public health concern, contributing to antimicrobial resistance, increased morbidity and mortality, and higher healthcare costs. This study aimed to evaluate antibiotic use in a tertiary hospital and assess the impact of Infectious Diseases and Clinical Microbiology consultations on prescribing practices.

Methods

On the study day, data were collected from all hospitalized adult patients receiving antibiotics. Variables included demographics, hospital ward, antibiotic name, route of administration, indication, dosage, frequency, duration, Infectious Diseases and Clinical Microbiology consultation status, and appropriateness of use.

Results

Of the 454 adult inpatients, 264 (58.2%) were receiving at least one antibiotic. Among them, 181 (68.6%) were treated empirically or with targeted therapy, while 79 (29.9%) received antibiotics for prophylaxis. The most commonly used antibiotic group was cephalosporins, used in 108 (40.9%) of cases. Respiratory tract infections were the most frequent indication. Antibiotic use was significantly higher in intensive care units compared to surgical and medical wards. Inappropriate antibiotic use was identified in 76 (28.8%) of the 264 patients. The rate of inappropriate use was significantly lower among patients who received consultations from the Infectious Diseases and Clinical Microbiology department. Conversely, inappropriate use was more frequent in surgical wards compared to medical wards and intensive care units.

Conclusion

Point prevalence surveys provide valuable insights into antibiotic prescribing patterns and support antimicrobial stewardship efforts. In line with findings from other centers, this study revealed that inappropriate antibiotic use was most common in surgical wards and for prophylactic purposes. Targeted interventions are needed to improve surgical prophylaxis practices.

## Introduction

Indications for antibiotic treatment can be categorised into three distinct classifications: the treatment of an identified infection, the treatment of a potential infection and prophylactic treatment. The treatment of a potential infection, or empiric treatment, is a frequently employed treatment method. However, in order to select the most appropriate antibiotic, it is essential that physicians accurately diagnose and identify the potential infectious disease. When selecting an empiric antibiotic, the frequency of the potential causative bacteria within the clinical picture and its variability with age and other host factors should be considered [1]. Prophylactic antibiotic use is defined as the administration of antibiotics with the objective of preventing the development of an infectious disease. Surgical prophylaxis is the most common method, and there are a limited number of medical prophylaxis indications.

Prior to the initiation of antibiotic therapy, it is incumbent upon the physician to consider several key questions. The pivotal question pertains to the indication of antibiotic therapy. The response to this question should be independent of the physician's personal concerns [2]. Infectious disease consultations play a significant role in guiding physicians from other specialties toward the most appropriate antibiotic selection, thereby supporting rational and effective antibiotic use [3].

The present study was conducted with the objective of evaluating the utilisation of antibiotics, the appropriateness of such use, and the effectiveness of infectious disease consultations in inpatients.

## Materials and methods

Study design

The study was conducted on March 5, 2025, at a tertiary Research and Practice Hospital with 546 adult beds, using the point prevalence method. All adult patients admitted to the wards prior to or at 8:00 a.m. on the designated study day and not yet discharged were included in the study. A data collection form was completed for each patient who used at least one systemic antibiotic. The form in question documented patient demographics and hospital admissions, in addition to the generic nomenclature of the antibiotic administered, the route of administration (parenteral, oral, rectal, or inhalation), the intended use (empirical for therapeutic purposes, targeted for therapeutic purposes, or prophylactic), the dosage, the frequency, and the duration of treatment. For patients receiving antibiotic treatment, the infections in question were classified as either community-acquired or hospital-acquired. The diagnostic group for each infection was also recorded according to anatomical site. The classification of prophylaxis was determined by its modality, categorised as either surgical or medical. The duration of surgical prophylaxis was grouped as follows: single dose, one day, or longer than one day. Furthermore, it was noted whether a consultation was requested from the Infectious Diseases and Clinical Microbiology (IDCM) Clinic and whether microbiological tests were performed. Infectious Diseases and Clinical Microbiology Clinic consultations are requested by the treating physician through the hospital's electronic medical record system.

The use of antibiotics without a valid indication, inappropriate dose, interval and duration, and the choice of broad-spectrum antibiotics when alternatives were possible were considered as inappropriate antibiotic use.

Information pertaining to the inpatient status of the subjects under investigation was obtained from the relevant information-processing records.

Statistics

Statistical analysis was performed using IBM SPSS Statistics for Windows, Version 27.0 (IBM Corp., Armonk, NY, USA). The chi-square test was used to compare categorical variables, and p values <0.05 were considered statistically significant. Odds ratios (OR) were calculated to determine the strength of the relationship between categorical variables.

## Results

On the day of the study, 454 adult patients were hospitalized. Among them, 264 (58.2%) were receiving at least one antibiotic. The mean age was 61.79 ± 18.04 years, and 134 (50.8%) were female. Among patients receiving antibiotics, 162 (61.4%) were on monotherapy, 95 (36.0%) on dual therapy, and seven (2.7%) on triple therapy.

Regarding indications, 181 (68.6%) received antibiotics for empirical or agent-specific treatment, and 79 (29.9%) for prophylaxis. Of those receiving prophylaxis, two (2.5%) received medical prophylaxis and 77 (97.5%) surgical prophylaxis. One patient (0.4%) was prescribed antibiotics for both treatment and surgical prophylaxis. In five (1.9%) cases, no indication for antibiotic use was identified. Prolonged duration was observed in 45 (58.4%) of the 77 patients who received antibiotics for surgical prophylaxis.

Cephalosporins were the most frequently prescribed antibiotic group, used in 108 (40.9%) of the cases. A complete list of antibiotics and their indications is presented in Table 1.

**Table 1:** Antibiotics in Use on the Study Day and Their Indications MP: Medical Prophylaxis, SP: Surgical Prophylaxis, ET: Empirical Treatment, DT: Definitive Treatment

Antibiotics	n=264	%	MP		SP		ET		DT	
			N	%	N	%	N	%	N	%
Cephalosporins	108	40.9	1	0.9	56	51.9	45	41.7	6	5.6
Ceftriaxone	62	23.5								
Cefazolin	37	14								
Cefuroxime	6	2.3								
Ceftazidime	1	0.4								
Cefixime	1	0.4								
Ceftibuten	1	0.4								
Beta-lactamase inhibitor + Penicillin combination	82	31.1	2	2.4	13	15.9	55	67.1	12	14.6
Piperacillin-Tazobactam	54	20.5								
Ampicillin-Sulbactam	22	8.3								
Amoxicillin-Clavulanic acid	6	2.3								
Carbapenems	40	15.2	0	0	0	0	29	72.5	11	27.5
Meropenem	37	14								
Imipenem	2	0.8								
Ertapenem	1	0.4								
Glycopeptide / Lipopeptide	35	13.3	0	0	0	0	19	54.3	16	45.7
Teicoplanin	23	8.7								
Vancomycin	10	3.8								
Daptomycin	2	0.8								
Quinolones	33	12.5	1	3.0	11	33.3	16	48.5	5	15.2
Ciprofloxacin	18	6.8								
Levofloxacin	11	4.2								
Moxifloxacin	4	1.5								
Nitroimidazoles	26	9.8	0	0	18	69.2	7	26.9	1	3.8
Metronidazole	15	5.7								
Ornidazole	11	4.1								
Macrolides	14	5.3	0	0	0	0	14	100	0	0
Clarithromycin	13	4.9								
Azithromycin	1	0.4								
Polymyxins	11	4.2	0	0	0	0	6	54.5	5	45.5
Polymyxin B	8	3.1								
Colistimethate sodium	3	1.1								
Clindamycin	6	2.3	0	0	3	50	2	33.3	1	16.7
Tetracyclines	6	2.3	0	0	0	0	2	33.3	4	66.7
Tigecycline	4	1.5								
Doxycycline	2	0.8								
Trimethoprim-Sulfamethoxazole	5	1.9	1	20	0	0	0	0	4	80
Linezolid	2	0.8	0	0	0	0	1	50	1	50
Aminoglycosides	2	0.8	0	0	0	0	0	0	2	100
Amikacin	1	0.4								
Gentamicin	1	0.4								
Fosfomycin	2	0.8	0	0	0	0	1	50	1	50
Rifampin	1	0.4	0	0	0	0	0	0	1	100

On the day of the study, 199 (43.8%) patients were in surgical wards, 194 (42.7%) in internal medicine wards, and 61 (13.4%) in intensive care units (ICUs). Antibiotic use was significantly higher in ICU patients compared to others (p = 0.003). The ward-based distribution of antibiotic use is shown in Table 2.

**Table 2:** Prevalence of Antibiotic Use in Intensive Care Units and Wards

	Intensive Care Unit	Medical Wards	Surgical Wards	p-value
Hospitalized patients	61	194	199	
Patients receiving antibiotics	47 (77%)	101 (52.1%)	116 (58.3%)	0.003

Among the 181 patients receiving antibiotics for treatment, the most frequent indications were respiratory tract infections (n = 79, 43.6%), followed by intra-abdominal infections (n = 26, 14.4%), and urinary tract infections (n = 19, 10.5%). Microbiological documentation was present in 51 (28.2%) of these patients. Hospital-acquired infections were identified in 97 (53.6%) and community-acquired infections in 84 (46.4%). When the 51 documented culture results were examined, it was seen that the majority of the growths were Gram-negative agents (n=32, 62.7%). The most frequently detected microorganism was Pseudomonas spp. (n=12, 23.5%). The most frequently detected microorganism among Gram-positive bacteria was S. aureus (n=8, 15.7%).

Among the 264 patients receiving antibiotics, 143 (54.2%) had prescriptions based on recommendations from the Infectious Diseases and Clinical Microbiology (IDCM) department.

The overall rate of inappropriate antibiotic use was 76 (28.8%). The most common reason was prolonged surgical prophylaxis. Detailed causes are listed in Table 3.

**Table 3:** Reasons for Inappropriate Antibiotic Use

Reasons	N = 76	%
Prolonged surgical prophylaxis	45	0.592
Use without indication	12	0.158
Inappropriate dosing	11	0.145
Unnecessarily broad-spectrum use	8	0.105

A significant association was identified between a consultation request from our clinic and the appropriateness of antibiotic use (p<0.001). The inappropriate antibiotic use was seven (4.9%) in patients who consulted at the IDCM clinic, while the inappropriate antibiotic use was 69 (57%) in patients who did not consult. The probability of improper antibiotic utilisation was found to be 26 times higher in patients who did not seek consultation (OR: 25.78; 95% CI: 11.12-59.75; p<0.001).

A significant relationship was also identified between the hospital wards where patients were admitted and the appropriateness of antibiotic use. Inappropriate antibiotic use was observed in 94 of 199 (47.4%) patients in surgical wards, 33 of 194 (16.8%) in internal medicine wards, and five of 61 (8.5%) in intensive care units. This variation was found to be statistically significant (p < 0.001).

## Discussion

Appropriate antibiotic use is defined as the administration of the narrowest-spectrum antibiotic that is required to meet the patient's needs, in appropriate doses, for appropriate durations, and by appropriate routes, when indicated. This approach has been demonstrated to be effective in preventing both the development of antibiotic resistance and the financial burden associated with treatment [2,4]. A range of studies conducted within our nation have reported that the utilisation of antibiotics among hospitalised patients varies between 36.2% and 63.2%. This high rate of antibiotic use renders appropriate use all the more crucial. When studies examining this topic are evaluated, the appropriate use rate is observed to range from 32% to 80% [5-7]. In the present study, consistent with the extant literature, the utilisation of antibiotics was observed in 264 (58.2%) of patients who were hospitalised. The appropriate utilisation rate of antibiotics is 188 (71.2%).

The utilisation of combination antibiotic therapy is recommended in order to ensure a comprehensive spectrum of activity and to forestall the emergence of resistance in infections caused by potentially resistant microorganisms. However, the prevailing guidelines do not advocate combination therapy due to the absence of compelling evidence substantiating its efficacy in cases where susceptibility to a preferred antibiotic is demonstrated [8]. In the present study, 162 (61.4%) of patients were receiving monotherapy. The majority of patients receiving combination therapy were admitted to intensive care units, and the utilisation of combination antibiotics is driven by the objective of efficacy against both resistant Gram-negative and Gram-positive bacteria.

The fundamental question that must be addressed prior to the initiation of antibiotic treatment is whether the administration of such medication is indicated [2]. In the present study, the utilisation of antibiotics was observed in five patients who did not demonstrate any indication for such treatment. Antibiotics are generally used for three main purposes: firstly, in the presence of a proven infection (i.e. targeted to the causative agent); secondly, empirically before a microbiological diagnosis is made; and thirdly, prophylactically [1]. Prophylactic antibiotic use is categorised into two distinct groups: surgical and medical. The predominant rationale for antibiotic utilisation in a hospital setting pertains to perioperative prophylaxis. It is estimated that antibiotic use related to surgical prophylaxis accounts for approximately 20% of total antimicrobial consumption in healthcare settings [9,10]. Surgical prophylaxis represents a particular area of concern with regard to the inappropriate use of antibiotics. Inappropriate antibiotics are frequently selected, and there is often excessive reliance on long-term antibiotic use, while the initiation of prophylaxis is sometimes initiated too early. In order to ensure appropriate use, prophylactic antibiotics should be administered either as a single dose or for a short period of time [11]. In the present study, 77 out of 79 patients (97.5%) who received prophylactic antibiotics were administered surgical prophylaxis, while only two patients received medical prophylaxis. Furthermore, an observation was made that prolonged surgical prophylaxis duration, which exceeded one day, was observed in 58.4% of patients receiving antibiotics for surgical prophylaxis. Prolonged surgical prophylaxis was identified as the most prevalent cause of inappropriate antibiotic use, accounting for 59.2% of cases (see Table 3). Consistent with the findings of this study, the most prevalent causes of inappropriate antibiotic use as documented in the extant literature are prolonged prophylaxis duration and unnecessary antibiotic therapy [5-7,12].

A plethora of studies have demonstrated that the rate of inappropriate antibiotic use in patients hospitalised in surgical wards is statistically significantly higher than in other wards [5,13,14]. As demonstrated in the present study, this phenomenon is associated with the utilisation of antibiotics that do not require approval [5]. As stipulated in the Health Practice Communiqué, the prescription of broad-spectrum antibiotics is contingent upon the approval of an IDCM specialist in the hospital in our country. Nevertheless, as a significant proportion of antibiotics employed for surgical prophylaxis are not encompassed within the restricted group, it has been asserted that current regulations are insufficient to impede inappropriate utilisation in this domain [14].

The distinction between hospital-acquired and community-acquired infections is of paramount importance in the empirical selection of antibiotics. Community-acquired infections frequently involve microorganisms that are less resistant to treatment. However, in the case of patients with hospital-acquired infections, a broader-spectrum treatment is required in view of the risk of infection with multidrug-resistant (MDR) pathogens [15]. Higher rates of antimicrobial resistance are observed in hospital-acquired infections than in community-acquired infections [16-18]. In the present study, 97 (53.6%) patients receiving antibiotic treatment were diagnosed with hospital-acquired infections, while 84 (46.4%) were diagnosed with community-acquired infections. Analysis of our culture results revealed that the majority of growths were Gram-negative agents (n=32, 55.2%). Pseudomonas spp. (n=12, 20.7%) and S. aureus (n=8, 13.8%), two microorganisms with a high prevalence in hospital-acquired infections, were the two most frequently detected microorganisms.

A meta-analysis of point prevalence studies of antibiotics conducted in Turkish hospitals revealed a median percentage of empirical antimicrobial use of 71% across 12 studies [19]. Despite the recommendation that microbiological samples should be obtained from the focus of infection in order to identify the causative agent prior to the initiation of empirical treatment, this is frequently disregarded. In the present study, 51 of the patients had microbiological documentation, and the rate of antibiotic use targeting the causative agent was found to be 28.2%.

The most prevalent antibiotic groups in hospital settings are third-generation cephalosporins and penicillins with beta-lactamase inhibitors [6,20-22]. In the present study, cephalosporins were the most frequently employed antibiotic group, accounting for 108 (40.9%) of cases (see Table 1). Antibiotic selection in our hospital is influenced by several factors: drug availability in the hospital pharmacy, cost to the patient or family when not covered by the hospital budget, internal treatment guidelines and the personal experience and preferences of the prescribing physician or surgeon.

In the present study, the utilisation of antibiotics in intensive care patients was found to be 77%, which was statistically significantly higher (p=0.003) in comparison to surgical and internal medicine clinics (see Table 2). This finding is consistent with the extant literature [10]. The high prevalence of MDR microorganisms and healthcare-associated infections in intensive care units is among the most significant factors contributing to the high antibiotic consumption in such units. Antimicrobial resistance and the frequency of infections observed in intensive care units are associated with numerous predisposing factors, such as advanced age, immunosuppression, prolonged hospitalization, intensive and broad-spectrum antibiotic use, and increased invasive procedures [23]. Moreover, a number of issues have been identified as factors that hinder the efficacy of antibiotic management within intensive care units. Two significant issues are identified as being of particular concern: firstly, diagnostic uncertainty and, secondly, clinicians' fear of inadequate coverage of the causative pathogen, especially in critical situations such as sepsis. These two factors have been demonstrated to increase the utilisation of multiple and broad-spectrum antibiotics, thereby reinforcing clinicians' hesitations regarding de-escalation and discontinuation of antibiotics [24]. Despite the intensive use of antibiotics, inappropriate antibiotic use in intensive care units is less common than in other clinics, at 8.5% (five of 61) in this study. This may be attributed to the regular monitoring of patients by an infectious disease specialist within the scope of the Infection Control Committee.

A point prevalence study conducted by the European Centre for Disease Prevention and Control (ECDC) on healthcare-associated infections and antimicrobial use, with participation from 23 countries, found that lower respiratory tract infections (29.2%) were the most common indication for antibiotic use for treatment, followed by urinary tract infections (14.9%), systemic infections (bacteremias) (14.7%), and soft tissue infections (14.2%) [25]. An antibiotic point prevalence study conducted in our country found that antibiotics were initiated with the highest frequency for respiratory tract infections (33.9%), urinary tract infections (19.1%), and skin and soft tissue infections (18.5%) [26]. In the present study, an examination was conducted of diagnoses for which patients received antibiotic treatment. The most prevalent diagnoses were respiratory tract infections 79 (43.6%), followed by intra-abdominal infections 26 (14.4%) and urinary tract infections 19 (10.5%).

The inappropriate use of antimicrobial agents has been demonstrated to accelerate the selection of resistant microorganisms, increase the incidence of adverse effects during treatment, and result in increased healthcare costs [14,27]. Infectious disease specialists make significant contributions to the appropriate use of antibiotics by providing guidance to physicians from other branches, particularly during the treatment process, through the consultation services they provide [27]. In the present study, it was observed that 143 (54.2%) of patients receiving antibiotics received them with the recommendation of an IDCM consultation. The rate of inappropriate antibiotherapy use in patients without consultation (69, 57%) was found to be significantly higher than in patients with consultation (seven, 4.9%) (p<0.001). The positive effect of IDCM consultation on appropriate antibiotic use is also supported by similar studies conducted in our country and abroad [13,28,29].

This study has several limitations. Being a single-center and point prevalence design limits the generalizability of the findings. The retrospective collection of data from medical records carries a risk of missing or inaccurate information, particularly regarding antibiotic use. These limitations should be considered when interpreting the results. Nevertheless, the study provides valuable insights into antibiotic prescribing practices and contributes to the development of antimicrobial stewardship strategies.

## Conclusions

In conclusion, routine antibiotic prevalence studies are effective in reviewing current practices and improving antibiotic management. In the current hospital, primary examples of inappropriate antibiotic use, similar to the literature, were observed in surgical wards and in the context of prophylactic use. Efforts should focus on improving surgical prophylaxis practices. Furthermore, seeking support from Infectious Disease specialists regarding antibiotic selection is recommended, as their consultation can significantly contribute to more appropriate prescribing and improved management outcomes.
